# Institutional Objection to Voluntary Assisted Dying in Victoria, Australia: An Analysis of Publicly Available Policies

**DOI:** 10.1007/s11673-023-10271-6

**Published:** 2023-07-10

**Authors:** Eliana Close, Lindy Willmott, Louise Keogh, Ben P. White

**Affiliations:** 1 Australian Centre for Health Law Research, Faculty of Business and Law, Queensland University of Technology, GPO Box 2434, Brisbane, QLD 4001 Australia; 2 Centre for Health Equity, Melbourne School of Population and Global Health, University of Melbourne, Melbourne, VIC 3010 Australia

**Keywords:** End-of-life issues, Euthanasia, Suicide, assisted, Health policy, Conscientious refusal to treat

## Abstract

**Background:**

Victoria was the first Australian state to legalize voluntary assisted dying (elsewhere known as physician-assisted suicide and euthanasia). Some institutions indicated they would not participate in voluntary assisted dying. The Victorian government issued policy approaches for institutions to consider

**Objective:**

To describe and analyse publicly available policy documents articulating an institutional objection to voluntary assisted dying in Victoria.

**Methods:**

Policies were identified using a range of strategies, and those disclosing and discussing the nature of an institutional objection were thematically analysed using the framework method.

**Results:**

The study identified fifteen policies from nine policymakers and developed four themes: (1) extent of refusal to participate in VAD, (2) justification for refusal to provide VAD, (3) responding to requests for VAD, and (4) appeals to state-sanctioned regulatory mechanisms. While institutional objections were stated clearly, there was very little practical detail in most documents to enable patients to effectively navigate objections in practice.

**Conclusion:**

This study demonstrates that despite having clear governance pathways developed by centralized bodies (namely, the Victorian government and Catholic Health Australia), many institutions’ public-facing policies do not reflect this guidance. Since VAD is contentious, laws governing institutional objection could provide greater clarity and regulatory force than policies alone to better balance the interests of patients and non-participating institutions.

## Introduction

Reflecting trends internationally, Australia has recently experienced a wave of voluntary assisted dying (VAD) reform. There is significant community support for VAD in Australia (Cartwright and Douglas 2017). In the last six years all six states have passed VAD legislation, and federal legislation that prohibited the territories from passing VAD laws has been repealed (Table 1). A near-universal feature of assisted dying laws are provisions that protect the right of individual health practitioners to refuse to participate in VAD due to a conscientious objection (Haining and Keogh 2021). However, a controversial issue gaining prominence is non-participation by healthcare entities, what we have described as an “institutional objection” (White et al. 2021) or, for some, an “institutional conscience-based objection” (Wolfe and Pope 2020).

**Table 1:** Status of Australian voluntary assisted dying laws in June 2023

Jurisdiction	Status
Jurisdictions that have passed VAD legislation	
Victoria	The *Voluntary Assisted Dying Act 2017* (Vic) was passed in November 2017 and took effect on June 19, 2019, after an eighteen-month implementation period.
Western Australia	The *Voluntary Assisted Dying Act 2019* (WA) was passed in December 2019 and took effect on July 1, 2021, after an eighteen-month implementation period.
Tasmania	The *End-of-Life Choices (Voluntary Assisted Dying) Act 2021* (Tas) was passed in March 2021. The law commenced on October 23, 2022, after an eighteen-month implementation period.
South Australia	The *Voluntary Assisted Dying Act 2021* (SA) was passed in June 2021 and commenced on January 31, 2023.
Queensland	The *Voluntary Assisted Dying Act 2021* (Qld) was passed in September 2021 and took effect on January 1, 2023, after a fifteen-month implementation period.
New South Wales	The *Voluntary Assisted Dying Act 2022* (NSW) was passed in May 2021 and will take effect on November 27, 2023, after an eighteen-month implementation period.
*Jurisdictions without VAD legislation*	
Northern Territory	The Northern Territory passed the *Rights of the Terminally Ill Act 1995* in May 1995 (taking effect on July 1, 1996). The Act was nullified in 1997 when the Australian Parliament passed the *Euthanasia Laws Act 1997* (Cth), which prohibited the territories from legislating on VAD. The *Restoring Territory Rights* *Act* *2022* (Cth), which took effect December 13, 2022, now enables the territories to pass VAD legislation should they choose to do so.
Australian Capital Territory	As a territory, the Australian Capital Territory was prohibited from legislating on VAD by the *Euthanasia Laws Act 1997* (Cth). With the passage of the *Restoring Territory Rights Act 2022* (Cth), a voluntary assisted dying bill is expected to be introduced into the ACT Legislative Assembly in the latter half of 2023.

An institutional objection occurs when an entity such as a hospital, aged-care facility, retirement village, or hospice refuses to provide or facilitate a lawful health service on the basis of its values (Sumner 2019). Institutional objections are commonly—though not exclusively (Sumner 2021; Queensland Law Reform Commission 2021)—claimed by faith-based institutions (Wolfe and Pope 2020; Wicclair 2012; Kirby 2021), particularly Catholic ones, which have objected to a wide range of legal health services, including abortion, treatment of miscarriages, contraception, and VAD (Wicclair 2012; Gilbert 2020; Chavkin, Leitman, and Polin 2013). Institutions may object to a range of activities surrounding a particular health service, including providing information, consulting about treatment options, making a referral, and providing the treatment or procedure itself.

There is significant ethical debate about the extent to which institutional objection should be permitted, and on what basis. Opponents of allowing institutional objection argue that unlike individuals, institutions do not have awareness and capacity, therefore they cannot have a conscience or possess moral or ethical objections (Wicclair 2012; Annas 1987). Another argument against accommodating institutional objection is that it can interfere with an individual’s autonomous right to access lawful health services (Kirby 2021). This is viewed as particularly problematic when a health or care provider is publicly funded. Additionally, from a system perspective, unlike individual conscientious objection, institutional objection can considerably compromise the public’s ability to receive services and “will almost always wipe out access for huge numbers of people because institutions serve huge numbers of people” (Gilbert 2020; Flynn and Wilson 2013). A further argument against institutional objection is that it can interfere with the conscience rights of practitioners who do not share the institutional position and wish to assist patients to access these lawful services (McDougall et al. 2021). In contrast to these arguments, proponents for recognizing institutional objection argue that an institution with a genuine mission, such as a religious hospital or aged care facility, possesses a unique identity that is analogous to the conscience of an individual health professional and should be protected (Wicclair 2012; Durland 2011). Another related argument is hospitals (or other entities) with religious affiliations are more attractive to staff and patients or residents who choose these entities because they share their values (Bussey 2018). Some also argue that institutional objection is part of an entity’s right to self-governance and should be protected provided there are legitimate reasons for the refusal to participate (Shadd and Shadd 2019; Card 2017).

There is growing evidence that institutional objections occur in practice and impact patients seeking lawful health services. One reason for this is the ubiquity of Catholic health facilities. In Australia, as in other countries (Catholic News Agency 2010), Catholic institutions receive a mix of public and private funding (Duckett and Willcox 2015), and provide a significant proportion of health, residential aged care, and palliative care services. Catholic Health Australia (the largest non-government association of hospitals, aged care, and community care services) estimates its members provide 15 per cent of hospital-based health services in Australia (30 per cent of private hospital care and 5 per cent of public), 12 per cent of aged care facilities, and 20 per cent of home care for older persons (Catholic Health Australia 2021). Around one in six patients in Australia are cared for in Catholic hospitals. In the context of end-of-life care, Catholic Health Australia members also provide a significant proportion of palliative care in Australia, servicing 13 per cent of palliative care related hospitalizations and 73 per cent of private inpatient palliative care beds and providing both public and private community-based palliative care (Catholic Health Australia 2019).

The prevalence of Catholic health facilities means that institutional objections can significantly impact access to health services. In the case of abortion, Stark (2013) reported that in Victoria, “Catholic-run services—which represent a third of the state’s public hospitals—do not offer terminations at all” (Stark 2013). This not only reduces access to a common, safe health service, but is particularly problematic for pregnant women diagnosed with a fetal abnormality at a Catholic hospital, who may need to be discharged to a public hospital participating in abortion to continue their care (de Crespigny and Savulescu 2008; Freedman, Landy, and Steinauer 2008). Similar access issues arise for VAD. Since VAD has been operational only relatively recently in most Australian states (Table 1), evidence is still emerging about the impact of institutional objection in practice. Catholic Health Australia has indicated its members will not participate in VAD, as euthanasia is contrary to the Catholic Code of Ethics (Catholic Health and Aged Care Services 2019).^1^ In Victoria (the first state to legalize VAD), institutional objections have been reported in a range of settings including hospices, palliative care units, and aged care facilities (White et al. 2023a; White et al 2023b; White et al. 2021; Waran and William 2020; Cunningham 2021; Corke and Bhatia 2022). The first Australian study of this issue found that institutional objections in Victoria resulted in delays in accessing VAD, transfers to other locations, and in some cases forced patients to choose between accessing VAD and receiving palliative or other care (White et al. 2023a). This caused patients and their family caregivers to experience anger, fear, and distrust. Similarly, accounts from Canada (where medical assistance in dying (MAiD) has been legal since 2016) demonstrate significant patient harms resulting from forced MAiD transfers including additional pain and suffering, financial costs, and a loss of decision-making capacity (and therefore access to MAiD) when heavy sedation or pain relief is required to facilitate a transfer (White et al. 2021; Gilbert 2020). Access issues may be compounded in rural or remote areas where access to healthcare is already constrained, or where an alternative location is unavailable. Transfers may cause more significant distress when the facility is a person’s place of residence, for example, a hospice or aged care facility (Queensland Law Reform Commission 2021).

Given the ethical controversy and the negative impacts of institutional objection on patients, debate has arisen about whether institutional objection should be regulated by law (White et al. 2021) or left to policies, whether at a governmental, organizational, or institutional level (Queensland Law Reform Commission 2021). There have been various approaches to legal regulation across jurisdictions (Queensland Law Reform Commission 2021). For example, in the United States, institutional conscience clauses are enshrined in law and typically grant broad opt-out rights (what Wicclair (2012) calls in the context of individual conscientious objection a “conscience absolutism” stance). In contrast, in Belgium a 2020 amendment (recently upheld) (European Institute of Bioethics n.d.) effectively bars healthcare institutions from objecting (what Wicclair (2012, 2019) calls “non-toleration” or “the incompatibility thesis”). In Australia, three out of six jurisdictions with VAD laws (Queensland, South Australia, and New South Wales) include institutional objection provisions, which take a “reasonable accommodation” (Wicclair 2012) approach and attempt to balance patient and institutional interests. In the other three Australian states that have legalized VAD (Victoria, Western Australia, and Tasmania), as in other international jurisdictions (Gilbert 2020), the law does not contain any provisions on institutional objection (*Voluntary Assisted Dying Act 2017* (Vic); *Voluntary Assisted Dying Act 2019* (WA); *End-of-Life Choices (Voluntary Assisted Dying) Act 2021* (Tas)). In these jurisdictions, policy on institutional objection is therefore potentially the most significant source of regulation.

This study set out to describe and analyse publicly available policies expressing an institutional objection to VAD in Victoria, Australia. As the first state in Australia to legalize VAD, data is emerging about how it is operating in practice (Willmott et al. 2021; Voluntary Assisted Dying Review Board 2021; Rutherford 2020) but little is known about how institutional objection is approached there. In Victoria, a significant amount of healthcare is provided in public and private religious institutions and there is no specific legal restraint on institutional conscientious objection. Therefore, this paper investigates the content of publicly available policy documents expressing an institutional objection in Victoria, asking:

*What is the nature of VAD policy documents expressing an institutional objection in Victoria, what guidance do they provide, and what are the implications of this guidance for patients or residents seeking VAD?*

The findings in this paper are instructive for international jurisdictions debating VAD laws and for ongoing consideration of the regulation of VAD in Australia, as it provides a case study to describe the impact of leaving institutional objection to policies, rather than legislation. It also provides insights on the development of policy in this challenging area of practice.

## The Victorian Policy Position

The Victorian Government policy position on VAD in institutions provides context for the policy analysis in this paper. As noted in the introduction, the Victorian VAD Act expressly allows individual registered health practitioners to refuse to participate in all aspects of VAD (*Voluntary Assisted Dying Act 2017* (Vic)) but is silent on institutional objection. This issue is instead addressed by policies issued by the Victorian government and by institutions themselves (Close, Willmott, and White 2021).

In the eighteen-month implementation period prior to the *Voluntary Assisted Dying Act 2017* (Vic) taking effect, the Victorian Government Department of Health developed a suite of VAD resources directed at health services (Table 2), available from a single webpage on the Department of Health’s website (Department of Health Victoria 2019). These sit alongside other Department of Health resources directed at health practitioners and at the community and health consumers. The key health service policy addressing institutional objection is the *Voluntary assisted dying model of care pathways for health services* (“*Department of Health Model of Care Policy*”), issued in January 2019 after consultation with stakeholders. The policy instructs organizations to consider three optional “pathways”:

- **Pathway A**—Single service (health services that are willing and capable of providing VAD in their facilities);
- **Pathway B**—Partnership service (institutions that will support and facilitate VAD, but require partnership with other organizations or general practitioners (GPs) to provide the service); or
- **Pathway C**—Information and support service (health services that will not provide VAD, either because of resource constraints or moral opposition).

The *Department of Health Model of Care Policy* characterizes institutional objection as an issue of corporate self-governance. It indicates that health services may refuse to participate in VAD for conscience-based reasons and operational ones. The policy instructs institutions to assess their context and choose the “most appropriate” pathway, based on a range of factors including the health service’s existing provision of end-of-life care, workforce capacity, and presence of health practitioners willing to participate.

**Table 2:** Policy guidance and information about voluntary assisted dying for health services from the Victorian Government Department of Health website (Department of Health. Victoria 2019)

Title	Date	Length (pages)	Brief description
*Preparing for voluntary assisted dying*	Undated (context suggests 2018)	9	Preliminary guidance to assist health services make decisions about their degree of involvement in VAD. Provides resources to help health services prepare for the commencement of the Act, including a decision-making diagram to help health services determine their level of involvement, a readiness checklist, sample workforce survey questions, and a PowerPoint presentation for education.
*Health service participation in voluntary assisted dying*	August 2018	2	An overview of key considerations for health services as they prepare for the Act to come into effect. Indicates models of care are being developed. Notes that health services are not required to refer a patient requesting VAD to someone who will assist them.
*Voluntary assisted dying model of care pathways for health services (“Department of Health Model of Care Policy”)*	January 2019	14	Guidance for those responsible for implementing VAD in their health service, which aims to promote consistent care pathways across Victoria. It sets out three high-level pathways, informed by consultation with stakeholders, including process maps. Sets out high-level considerations that service providers should consider. Provides several recommendations regarding workforce preparation and education.
*Voluntary assisted dying safety and quality guidance for health services*	January 2019	47	Guidance to support implementation of VAD that is consistent with the Australian Commission on Safety and Quality in Health Care’s framework. Includes detailed questions for health services to consider and actions to ensure VAD obligations are integrated into existing policies and processes in a way that is consistent with the framework. Includes information on accreditation standards as they relate to VAD.
*Voluntary assisted dying guidance for aged care providers*	March 2019	7	Describes the three pathways in the *Department of Health Model of Care Policy* and sets out high-level considerations that aged care service providers should consider in determining the most appropriate model of care. Indicates that the Department of Health expects most aged care providers will adopt Pathway B (partnership service) or Pathway C (information and support service). Includes process diagrams.
*Voluntary assisted dying considerations in end-of-life care*	March 2019	7	Guidance for executives, managers and senior staff who are responsible for the systems and processes regarding VAD. Emphasizes that health services must ensure that end-of-life care is consistent with *Victoria’s end of life and palliative care framework* and provides general guidance to further this.
*Health service policy guidance for voluntary assisted dying*	April 2019	6	Intended to help health services who have chosen Pathway C (information and support service) develop local policies to respond to patients who request information about or access to VAD. Includes a policy template which suggests all requests for information about, or access to, voluntary assisted dying should be referred to the Statewide Voluntary Assisted Dying Care Navigator Service.

An institution that refuses to participate in VAD will usually fall under Pathway C. The *Department of Health Model of Care Policy* sets out recommendations for institutions that adopt this pathway. Regarding requests for VAD, the policy states all health services should be prepared to respond to requests for information about, or access to, VAD. It instructs institutions to “consider how voluntary assisted dying is incorporated into end of life care … [including] … the process for maintaining existing therapeutic relationships …” and recommends that each health service develop “a person-centred process for managing requests for voluntary assisted dying that provides clinical support and guidance to all health practitioners within the service” (Department of Health. Victoria 2019). Regarding referral, the policy does not require a health service to refer the patient to a VAD practitioner, but a health service must not “inhibit a person’s access to treatment.” The policy suggests health services should inform the patient “as soon as practicable that they will not assist them” and should nominate a VAD contact within the organization. The policy also states that objecting institutions may choose to refer individuals to its Statewide Voluntary Assisted Dying Care Navigator Service (“Care Navigators”). As with the other recommendations in the policy, this is framed permissively and is not mandatory. The *Department of Health Model of Care Policy* states:

Where health services will not facilitate or provide access to voluntary assisted dying, the person **can be provided** with details of the voluntary assisted dying care navigator … [emphasis added] (Victorian Department of Health 2019)

There is very limited data on the pathways that healthcare institutions in Victoria have adopted. One public tertiary hospital that adopted Pathway A has published its processes (Booth, Eleftheriou, and Moody 2021). McLaren and Mewett (two medical practitioners from Victoria who have provided VAD since the legislation commenced) report anecdotally that most health services in Victoria have adopted Pathway C (information and support service) (McLaren and Mewett 2021).

## Methodology

This study builds on a comprehensive analysis of policies (Close, Willmott, and White 2021), which sought to identify the prevalence and content of publicly available documents that aim to regulate VAD in Victoria. As discussed in the broader study, there is no single definition of “policy” in health policy research (Close, Willmott, and White 2021). This research focused on policies in the form of institutional and professional guidance, which represent “declarations of the organizations’ deeply-held values” (Goodridge 2010) and a framework for decision-making (Silvius, Memon, and Arain 2019). Regulation was construed broadly, using Parker and Braithwaite’s definition as aiming to “steer” or influence the “flow of events” or behaviour (Parker and Braithwaite 2003). Relying on this broad conception of regulation and literature on institutional end-of-life policies, we defined a “policy” as

- a written document (including in electronic form, written document (including in electronic form, such as a PDF, Microsoft Word document, or a webpage)
- issued by a government, institution, or organization (rather than a single individual), and
- that aims to steer the flow of events and behaviour through:
  - providing information,
  - issuing guidance, or
  - advancing a vision, outcome, or standard (Close, Willmott, and White 2021).

Policies were located using Google and health research databases (Scopus, PubMed) between May 13 and July 10, 2020, using these keywords: voluntary assisted dying, VAD, assisted dying, euthanasia, physician assisted suicide, assisted suicide, palliative care, guidelines, guidance, policy, position, statement, Victoria, government. Entities’ websites were checked periodically for updates in April 2021, on July 28, 2021, and on May 26, 2022. Any policy updates were noted and checked for any changes in substance.

The larger analysis identified policies issued by the government (including the *Department of Health Model of Care Policy* discussed above), health professional organizations, and health institutions. For this narrower study, we isolated those policies from the larger analysis that expressed an institutional objection to VAD. Institutional objection was defined as *an entity’s refusal to provide at least some aspect of VAD (including the provision of information) on the basis of integrity, morality, or values.* Policies addressing an in-principle objection to VAD generally prior to the VAD Act passing were excluded as they were not aiming to regulate (i.e., steer the flow of events or behaviour regarding institutional objection in practice).

The policies were analysed inductively using the framework method of thematic analysis (Gale et al. 2013). The lead author (EC) coded all the policies using NVivo 12 qualitative software (QSR International) and developed a preliminary list of themes. BW and LW then independently coded five policies each and EC, BW, and LW developed the final themes through iterative discussion. Once the policies were coded, they were charted into a matrix using Microsoft Excel, to facilitate comparison and assist in interpretation, in accordance with the framework method.

## Results

### Description of Sample

The broader study on which this research is based identified nineteen policies from eleven health entities (Close, Willmott, and White 2021). Fifteen policies (from nine entities) expressed an institutional objection to VAD and therefore were included in this study (Table 3). The entities that drafted these policies provide health, residential, and palliative care services and operate a wide range of facilities from public and private hospitals to residential aged care facilities. Eight of the nine objecting entities were explicitly faith-based with Banksia Palliative Care Service, a palliative care provider, being the exception. Seven of these faith-based institutions were Catholic (Cabrini, Calvary Health Care, Catholic Health Australia, Eastern Palliative Care, Mercy Health, St John of God Health Care, St Vincent’s Health Australia), and one was Jewish (Jewish Care) (Table 3). Most of the Catholic institutions are affiliated with Catholic Health Australia. Eastern Palliative Care is affiliated with the Catholic Order of Malta.

**Table 3:** Policies expressing an institutional objection to participating in the *Voluntary Assisted Dying Act* 2017 in Victoria, Australia

No.	Entity	Policy name	Length (PDFpages)	Date	Perceived audience	Type of document	Medical language	Ethical language	Religious language	Operational language
Catholic health care entities										
1	Cabrini	Cabrini's approach to end-of-life care	2	December 20, 2018	Public	Media release	✓	✓	✓	✘
2	Calvary Health Care	Position Statement on Euthanasia, Physician Assisted Suicide and Voluntary Assisted Dying	3	March 5, 2019	Public	Position statement	✓	✓	✓	✘
3	Calvary Health Care	Voluntary Assisted Dying FAQs	3	May 6, 2016	Public, staff	FAQs	✓	✓	✘	✓
4	Calvary Health Care	Voluntary Assisted Dying Bill 2017 Calvary's Position	9	August 15, 2017	Public, government, administrators	Position statement	✓	✓	✘	✘
5	Catholic Health Australia	Our enduring commitment to end of life care	2	February 2019	Public, administrators, staff	Brochure	✓	✓	✓	✓
6	Catholic Health Australia	CHA VAD Response Taskforce: Clinical Governance Recommendations	9	February 2019	Administrators, staff	Report	✓	✓	✘	✓
7	Catholic Health Australia	Joint Media Statement	4	June 19, 2019	Public	Media release	✓	✓	✘	✓
8	Eastern Palliative Care	Code of Ethics	8	November 10, 2020	Public, staff	Code of ethics	✓	✓	✓	✘
9	Eastern Palliative Care	Euthanasia Statement	2	September 28, 2015	Public, staff	Position statement on VAD	✓	✓	✘	✘
10	Mercy Health	Mercy Health’s response to Physician Assisted Suicide	3	July 21, 2017	Public	Media release	✓	✓	✓	✘
11	St John of God Health Care	Position statement on assisted dying	2	Undated	Public	Position statement on VAD	✓	✓	✓	✘
12	St Vincent’s Health Australia	Palliative and end of life care position statement	12	April 2017	Public, government, administrators, staff	Position statement on end of life care	✓	✓	✘	✘
13	St Vincent’s Health Australia	Position on Assisted Suicide and Euthanasia	2	Undated	Public	Brochure	✓	✓	✘	✘
*Non-Catholic health entities*										
14	Banksia Palliative Care Service	Voluntary Assisted Dying Position Statement	2	2020	Public	Position statement on VAD	✓	✘	✘	✘
15	Jewish Care	Position Statement: Voluntary Assisted Dying	2	April 2019	Public, staff	Position statement on VAD	✓	✓	✓	✓

Note: In June 2021, Catholic Health Australia’s website was redesigned and documents #5–7 are no longer available online. Calvary Health Care issued an updated position statement (#2) on November 11, 2021, and updated FAQs on February 28, 2022 (#3), which remain substantively similar to their previous iterations

The policies ranged in length from three paragraphs (Banksia Palliative Care Service) to twelve pages (St Vincent’s Health Australia) and took different formats (Table 3). Seven were labelled as “position statements,” four were information documents (i.e., brochures, reports, or frequently asked questions (“FAQs”)), three were media releases and one was a code of ethics. All the policies except one were oriented towards a public audience; the Catholic Health Australia *CHA VAD Response Taskforce: Clinical Governance Recommendations* (Table 3: Catholic Health Australia, #6) was aimed at administrators and staff implementing a response to VAD in Catholic Health Australia organizations. All documents used medical and ethical language, seven explicitly discussed religious values, and five used operational language (Table 3).

As well as the broad style and perceived audience of the polices, we identified four themes from the content of the policies: (1) scope of refusal to participate in VAD, (2) justification for refusal to participate in VAD, (3) how to respond to requests for VAD, and (4) appeals to state-sanctioned regulatory instruments.

### Theme 1: Scope of Refusal to Participate in VAD

A common component of policies was a description of the scope of the entity’s refusal to participate in VAD. The policies diverged in the level of detail they provided. Some simply made broad statements about non-participation without specifying the activities they will not be involved with. For example, Cabrini Health (a Catholic provider of acute and aged care services) states “we will not be participating in voluntary assisted dying for patients receiving care with us at our facilities or in their homes while under our care” (Table 3: #1). The policy does not elaborate further.

In contrast, several policies were more specific and explicitly indicated they will not facilitate or participate in VAD assessments or in the administration of the VAD substance (see, e.g., Table 3: Calvary Health Care, #2, #3; Catholic Health Australia, #5). Few policies explicitly addressed the provision of information. Jewish Care explicitly indicates it will provide information and refer patients to the Care Navigators (Table 3: #15). Catholic Health Australia indicates that organizations under its umbrella should train all staff to be aware that if asked about VAD they need to either provide information or “seek out an alternative staff member who can disclose information in a timely manner” (Table 3: #6). However, many of the Catholic policies falling under the Catholic Health Australia umbrella (see, e.g., table 3: Cabrini, #1; Calvary Health Care, #2; St John of God Health Care, #11) did not explicitly address whether or not they would provide information about VAD if a patient requested it.

Five of the fifteen policies stated explicitly that the institutional refusal to participate in VAD extends to employees (Table 3: Calvary Health Care, #3; Catholic Health Australia, #5–#7; Banksia Palliative Care Service, #14). For example, Catholic Health Australia (a national body representing a range of Catholic healthcare providers) indicated that none of the organizations that fall under its ambit (or their employees) are permitted to participate in VAD (Table 3: #5–#7). These policies presented the organization and its employees as having a unified position. A prohibition on employee participation was also implied in five policies (Table 3: Cabrini, #1; Calvary Health Care, #2; St John of God Health Care, #11; St Vincent’s Health, #12, #13), four did not address it (Table 3: Calvary Health Care, #4; Eastern Palliative Care, #8, #9; Mercy Health, #10), and in one it was unclear (Table 3: Jewish Care, #15). None of the policies expressly mention whether their employees are permitted to provide VAD in other institutions that are participating in VAD. Nor do any of the policies address whether they would permit willing health professionals from outside the organization to enter their premises to facilitate VAD.

### Theme 2. Justification for Refusal to Provide VAD

All entities except Banksia Palliative Care Service provided one or more justifications for their refusal to facilitate VAD. Three central justifications were: (1) VAD conflicts with religious values, (2) VAD is contrary to medical ethics/care, and (3) VAD detracts from palliative care.

First, several policies indicated that VAD is contrary to their religious values. For example, Catholic Health Australia and Calvary Health explain that their ethic is founded on “the longstanding Christian tradition of providing care, especially for the poor and vulnerable” (Table 3: #4; #5). St John of God Health Care “models its care on the healing mission of Jesus Christ” (Table 3: #11). Mercy Health (a Catholic Health Australia-affiliated provider of inpatient and community palliative care services, and residential aged care) states “life is sacred and not for individual self determination” (Table 3: #10). Similarly, Jewish Care notes it “is committed to upholding a fundamental tenet of Judaism that all human life is sacred” (Table 3: #15).

A second justification was that involvement in VAD is contrary to medical ethics. Calvary Health Care and Catholic Health Australia both reference the “Hippocratic ethic,” with the latter’s policy stating:

Our clinicians do not and will not intentionally inflict death on patients … We accept and act according to the Hippocratic commitment that these interventions are not medical treatments. (Table 3: #5).

A similar rationale was provided by other organizations affiliated with Catholic Health Australia, which indicate VAD is not considered “medical treatment” (see, e.g., Table 3: Calvary Health Care, #2; #3, #4), nor “part of end of life care” (Table 3: Catholic Health Australia, #6; Mercy Health, #10). A few documents from faith-based institutions emphasized this rationale and did not mention religion at all (Table 3: Catholic Health Australia, #7; Eastern Palliative Care, #9; Mercy Health, #10).

A third justification was that VAD detracts from palliative care. For example, Eastern Palliative Health (a publicly funded palliative care service affiliated with the Order of Malta and the Catholic Church) states it will not provide VAD because “this would compromise the ethos of Palliative Care in Australia” (Table 3: #9). Other policies suggested that VAD was almost always not necessary with adequate palliative care (see, e.g., Table 3: #10; #11; #12). For example, St John of God Health Care states:

We believe that comprehensive, excellent and compassionate end of life care … should be provided to all in need, so that no person need resort to contemplating assisted suicide. Our experience demonstrates that with the provision of high quality pain management, it is exceptionally rare for a patient to have significant discomfort at the time of death. (Table 3: #11)

### Theme 3. How to Respond to Requests for VAD

Another theme was guidance on how to respond to requests for information about or access to VAD. Some policies cited principles to guide how to respond to requests. For example, policies indicated they would respond “openly and sensitively” (Table 3: Cabrini, #1; Catholic Health Australia, #5) and “respectfully and compassionately” (Table 3: Catholic Health Australia, #7) and “without discrimination” (Table 3: Calvary Health Care, #2) to requests for VAD. Most of the policies were very high-level and did not include practical guidance about navigating these conversations or a process for responding to requests.

One exception was the Catholic Health Australia Taskforce document (Table 3: #6), which included detailed information about a tiered response to communication including discussion about end-of-life options and connections to other care pathways including GPs and community care. Catholic Health Australia indicates it has:

… developed clear guidelines and information for patients, their families and our staff to provide clarity around the end of life care available at our facilities. We have worked to ensure our staff are informed about those aspects of the Act that are relevant to them.

Catholic Health Australia also recommends that Catholic care facilities develop communication packages specific to their context. The Taskforce document (Table 3: #6) states public hospitals, private hospitals, and community and aged care settings should issue a position statement on VAD, a response to VAD policy, and consumer information regarding end-of-life care. Catholic Health Australia states that this consumer information should: 1) be provided to new residents in aged care facilities, 2) clarify the scope and extent of end-of-life services that are available “in organisations congruent with our position” and how they can be accessed, and 3) include the state health department VAD website and information line “which is the appropriate resource if a person wishes to seek out information of their own accord” (Table 3: #6).

Despite this Catholic Health Australia guidance, very few of the entities that fall under its umbrella reflect these processes and recommendations in their public-facing policies. Mercy Health only briefly mentions its system to respond to VAD inquiries (Table 3: #10). A couple of policies mention a system in place to facilitate transfers but do not provide details of what that system entails including what happens if there is no place to transfer the patient to. For example, Calvary Health Care indicated it has developed a policy entitled *Responding to Requests for Access to Voluntary Assisted Dying* and has a tiered response system in place (presumably developed from the Catholic Health Australia guidance) but does not provide public access to this policy or further details (Table 3: Calvary Health Care, #3).

Regarding transfers, only four of the fifteen policies explicitly address transfer or referring a patient for VAD (Table 3: Calvary Health Care, #3; Catholic Health Australia, #6, #7; Mercy Health, #10). For example, Catholic Health Australia states:

If people in our care wish to access “VAD” from other providers, our services will not impede them. We will provide release from care as well as transfer if they wish to access services elsewhere. (Table 3: #7)

### Theme 4. Appeals to State-Sanctioned Regulatory Mechanisms

Finally, some of the policies referred to state-sanctioned regulatory mechanisms, such as law and government policy, to support their position. For example, Banksia Palliative Care states it will not participate and “will maintain all boundaries as permitted and defined by law ….” Similarly, Jewish Care asserts:

The minimum, **legally compliant**, role an organisation must take is to ensure a person has access to information, if requested, and is referred to the statewide resources available to assist with their inquiry. (Table 3: #15, emphasis added)

Although this excerpt misstates the law (the VAD Act does not require organizations to provide information), it reflects the *Department of Health Model of Care Policy* Pathway C model of care (information and support service).

Other policies referred to having support from the Victorian Government. For example, Catholic Health Australia states that they have “an open line of communication” with the Department of Health, which is “fully informed of our response to this legislation” (Table 3: #7). Catholic Health Australia notes:

We appreciate their recognition of the promises made by the parliamentarians, that hospitals and other healthcare institutions will not be pressured into facilitating nor providing ‘VAD’. Our response aligns with relevant guidelines for non-participating services. (Table 3: #7)

## Discussion

### Synthesis of Policies

The purpose of this article was to describe and analyse publicly available policies developed in response to the *Voluntary Assisted Dying Act 2017* (Vic) which express an institutional objection to VAD. These policies all indicated an unwillingness to participate in or facilitate VAD; however, how policymakers chose to express and provide guidance to navigate their objections varied.

The nature of the justifications for institutional objection ranged from explicitly using religion through to focusing on medical ethics and the perceived incompatibility of VAD and palliative care. The justifications cited are congruent with those provided in a study from Belgium that reviewed VAD policies in Catholic institutions (Gastmans et al. 2006). Additionally, the range of non-religious reasons provided reflect those cited by individuals with a conscientious objection (Haining, Keogh, and Gillam 2021).

Notably, a few of the faith-based policies focused solely on medical justifications and did not mention religion, which could be to make the institutional objection more acceptable to staff, patients, or family members who do not share those religious beliefs. This is significant because a common feature of most of the policies is that they presented the institution and staff as having a unified position. This sits uncomfortably with surveys that suggest a significant proportion of Catholics in Australia support VAD (Cartwright and Douglas 2017). Moreover, there are a range of opinions about the relationship between VAD and palliative care, and in some countries, such as Canada and Belgium, the two practices are well-integrated (Cohen and Chambaere 2022). When employees do not share an institution’s views, healthcare professionals may experience frustration from being told they could not participate in VAD due to institutional objection (Brown et al. 2021; Stulberg et al. 2010; Stulberg, Jackson, and Freedman 2016). Clinicians might also miss out on the chance to develop desired skills in this area (McDougall et al. 2021). The characterization of a unified front in the policies does not allow for the expression of individual employees’ conscience or a process for when staff have a different view from their institution.

Another common feature was that the policies acknowledged the clinical reality that patients in their facilities may make requests for VAD. The policies indicate that patients will not be abandoned and that such requests demand a sensitive and compassionate response. However, the concrete issues of providing information about VAD and referral pathways in response to requests were not squarely addressed in most of the policies. Therefore, it is unclear from this sample whether the *Department of Health Model of Care Policy* recommendation that institutions ensure they are prepared to respond to requests for VAD has been followed. While a couple of the policies mentioned “coordinating safe transfer of care” if a patient or resident requested VAD, no further detail was provided, leaving considerable uncertainty about the process that would be followed. Catholic Health Australia sets out a comprehensive clinical governance procedure, but it is not clear from the other Catholic policies sampled whether these comprehensive pathways have been adopted at the institutional level.

### Gaps in Available Guidance and Implications for Patients

The analysis also demonstrated several gaps in the available policy guidance. First, many institutions do not have publicly available policies at all. A possible explanation is that institutions have internal policy but have not made this publicly available. This is the case with Calvary Health Care, which mentions its internal-facing policy. Calvary Health Care is a member of Catholic Health Australia and presumably relies on its umbrella clinical governance structure (Table 3: #6). However, since we conducted the initial analysis (Close, Willmott, and White 2021) the Catholic Health Australia Taskforce document is no longer publicly available, and it is unclear the extent to which organizations have adopted its guidance on clinical governance in internal policies.

If institutions have policies on this issue which are not made public, then this lack of transparency means that institutions’ objections are harder to navigate (White et al. 2023a). Prospective patients or residents cannot easily make decisions about what facilities to access when they are making decisions about their care. This might also be problematic for existing patients or residents who are seeking VAD but are unable to access information within the facility. Accessing an institution’s internal policy may require patients or residents knowing that it exists and feeling able to request a copy in a context of power and information asymmetry. While several documents indicate that organizations will never abandon their patients, it is not clear whether these policies would assist in ensuring that their patients or residents receive information about VAD if that is their choice. It is possible that this lack of external policy development is intentional as many conscientious objectors view providing information and making referrals as being “complicit” in provision of the service (Minerva 2017).

Second, while there was broad consensus in the policies that requests for VAD required a sensitive and compassionate response, it was not clear how such requests would be received or dealt with. In most of the policies there was no information about who in the organization to contact with queries about VAD. The *Department of Health Model of Care Policy* suggests all Pathway C organizations nominate a VAD contact within the organization. This was not apparent in the policies studied (though notably the Catholic Health Australia Taskforce policy suggested the development of a tiered clinical governance structure to handle VAD requests that nominates a single executive to be the VAD lead) (Table 3: #6). Other research has emphasized the importance of a “guiding person” to inform patients, families, and practitioners about how VAD requests are handled (White et al. 2023b; Lemiengre et al. 2010). While organizations studied may have such a nominated person, there is an absence of these details in the public-facing policy documents. This does not assist patients in navigating the institutional objection held by faith-based entities that provide health services and residential aged care services.

Third, another significant gap (related to the second) in most of the policies was a lack of what Lemiengre et al. describe as a “practical procedural approach” (Lemiengre, Dierckx de Casterlé, Denier, et al. 2008a, b; Lemiengre et al. 2014). A procedural approach provides step-by-step guidance to steer health professionals through processes such as responding to VAD requests. The Catholic Health Australia Taskforce document contemplates such a structure and indicates “we recommend all services work to establish tiered competencies across their systems” which includes staff either providing basic information about VAD or seeking out an alternative staff member to provide this. The Calvary Health Care FAQ document (Table 3: #3) and Mercy Health document (Table 3: #10) indicate the organization has adopted this tiered system (though does not provide detail about what this entails). The other organizations’ policies do not address this.

A potential reason for this gap in practical guidance could be that these documents were produced relatively early in the development of the VAD system in Victoria before institutions were familiar with the clinical scenarios that would arise in practice or before they had locally integrated Catholic Health Australia procedures. Another possible explanation, noted above, may be that the public-facing guidance is intended to be vague so that the service is not seen to be “complicit” in helping patients gain access to VAD (Minerva 2017). However, the lack of a practical procedural approach means that the Department of Health’s Pathway C recommendation to have a “person-centred process for managing requests” is missing from most of the documents (the Catholic Health Australia Taskforce document being an exception). Additionally, there are other practical issues that are unanswered. While a couple of the documents mentioned they would facilitate a transfer if a patient requested VAD, the documents are silent on what would happen if there was no place to transfer the patient to. There is also no detail about the VAD Care Navigators or the Victorian Government information website as resources. It is possible that internal processes exist to navigate these, but aside from the Catholic Health Australia document these were not explained.

### Implications for the Regulation of Institutional Objection

This study has broader implications relating to two key issues: (1) to what degree should institutional objection to VAD be permitted, and (2) what form should the regulation of institutional objection take?

On the first issue, there are three primary policy positions that a government could take:

- Conscience absolutism (allow institutional objection without constraints)
- Compromise or reasonable accommodation (allow institutional objection but impose some constraints)
- Non-toleration (institutional objection is prohibited) (White et al. 2021; Wicclair 2012)

The current Victorian policy position has led to the problems articulated because it arguably permits conscience absolutism, or a very weak form of compromise, at least in practice, by leaving access to VAD up to individual institutions to decide. The *Department of Health Model of Care Policy* and its accompanying suite of resources for health services (Table 2) suggest best practices and provide recommendations aiming to standardize practice. The minimum recommended obligation for objecting institutions under Pathway C is that organizations should provide information to patients seeking VAD. However, there is no obligation to provide information, let alone to take steps such as referring or transferring a patient. As a result, institutions have broad latitude to determine their approach to VAD as a matter of institutional self-governance (White et al. 2021). Our analysis found that some institutions’ documents cited the Department of Health policy position to reinforce their stance (theme 4).

To better protect patients from the harms articulated in the introduction (White et al. 2023a), the Department could have reached a policy position of non-toleration or reasonable accommodation that either (a) requires institutions to provide VAD (ie non-toleration); (b) require institutions to take active steps to transfer a patient and, if not possible, allow access at a facility (reasonable accommodation); or (c) some other version of compromise (White et al. 2021). If this was stated clearly in policy and the Department of Health embedded regulatory structures to ensure this is complied with (for example, by tying public funding or accreditation to certain minimum obligations regarding VAD), this would better balance the interests of patients with those of institutions.

This study also has implications regarding the second issue, namely once a government has settled on a policy position, what form the regulation of institutional objection should take. This study highlights why law, with its coercive force, has a greater ability to advance a reasonable accommodation or non-toleration approach in practice. Since institutional objection is a contentious issue with strongly held views from a variety of perspectives, it may require a stronger regulatory force to ensure compliance by institutions. Given that institutions are inherently more powerful than individual patients (who in this context are terminally ill and suffering), law is a better mechanism than policy to implement protections for patients in this contested setting (White et al. 2021). Law also has greater potential than policy to promote a consistent and certain approach across diverse healthcare settings. In recommending a legislative framework to underpin the regulation of institutional objection, the QLRC succinctly highlighted why such an approach is preferable:

Legislation, supported by more detailed policy statements, informs individuals and entities of the basic ground rules by which their respective rights and interests are reconciled and the process which applies. An individual can rely on such a legislative statement to compel an entity to respect the individual’s rights. Equally, an entity can rely on the law to explain its rights and obligations if a patient or resident insists on being provided with services that the entity is not obliged by law to provide. (494)

Looking at both issues together, this study highlights how the Victorian regulatory structure (with a policy position that preferences the interests of institutions and an absence of legislation on institutional objection) has resulted in a variety of different policy approaches and gaps in existing publicly available guidance. Whatever model of regulation is adopted, law and policy should clarify a number of important questions regarding the relative position of patients and institutions. The Queensland Law Reform Commission discussed at pages 459–460 of its report (Queensland Law Reform Commission 2021) (paraphrased) that these include:

- Does an entity that does not provide access to VAD have to refer a patient seeking VAD to another medical practitioner or to a Care Navigator?
- Does an entity have the right to hinder access to VAD (for example, by refusing entry to a medical practitioner who wishes to enter a facility to do an assessment or a pharmacist who is delivering the VAD substance)?
- Are there any circumstances when it is not reasonable to transfer a patient for VAD, for example, when the transfer would cause the patient intolerable pain and suffering or compromise their decision-making capacity? And if so, would VAD be available in the facility for such patients or does this inability to transfer mean that VAD cannot be accessed?

The results of this policy analysis do not provide clear answers to these wider normative questions. What it does reveal, however, is that the current policy-based approach in Victoria risks an ad hoc approach to each issue. This leaves patients (and health professionals external to a facility that may seek to facilitate VAD) without a clear sense of whether VAD may be available and on what conditions.

## Strengths and Limitations

This study is the first to examine publicly available policy statements expressing an institutional objection to the *Voluntary Assisted Dying Act 2017* (Vic). Although representing only one Australian jurisdiction, the Victorian approach provides a case study for other Australian states and international jurisdictions seeking to regulate the increasingly vexed issue of institutional objection. While the study did not capture internal policies, publicly available statements are an important aspect of information accessible to patients, residents, external health professionals, and the public. They are also often the basis for locally developed guidance.

More research is needed to determine which healthcare institutions and aged care facilities have policies on VAD, how these policies were developed, and how they are being implemented. This could involve survey methodology to elicit internal policies, akin to studies conducted in the Netherlands and Belgium (Gastmans et al. 2006; Lemiengre et al. 2007; Lemiengre et al., 2008a). More research is also needed on the extent to which health professionals, patients, and the public are aware of institutional objection policies and how these policies impact healthcare and access to VAD. More research will also be needed to examine how these statements evolve over time and to examine the policy environment in other jurisdictions where VAD is legal (both those with institutional objection laws and those without).

## Conclusion

In the six-year period since the *Voluntary Assisted Dying Act 2017* (Vic) was passed in Victoria, VAD reform has swept Australia (Table 1). By the end of 2023, 98 per cent of the Australian population will live in a jurisdiction where VAD is a legal option (Australian Bureau of Statistics 2021). A range of other international jurisdictions including New Zealand, New Mexico (USA), Austria, and Spain have also all recently passed VAD laws, with others considering it. We offer several recommendations that flow from this study, which may be of significance for jurisdictions, both in Australia and across the world, which are considering legalizing assisted dying or are implementing or reviewing existing legislative regimes.

First, governments should have a clear policy position on the balance between institutional objection and patient access. This position should be informed by ongoing research about the frequency of such objections, where they are occurring, the nature of practices objected to, and the extent to which patients, residents, and health professionals are negatively impacted, as well as an understanding of community expectations. While the policy position taken is ultimately up to governments, policymakers should appreciate the potential implications of the power imbalance between institutions and patients (White et al. 2023a, b). Failing to have a clear policy position or having a policy that permits conscience absolutism will cause harms to patients seeking VAD (White et al. 2021).

A second recommendation is that once the government’s policy position is settled, ideally it should be enshrined in legislation, rather than solely in policy. The two benefits of legislation rather than policy alone are that it promotes a clear and consistent approach and can compel behaviour. However, legislation alone is not sufficient, given the number of stakeholders involved in this contested space. The optimal model for regulating institutional objection involves having clear, coherent guidance from a variety of regulatory sources.

Third, regardless of the policy position and regulatory structure adopted, governments and institutions should foster more transparency about their position on institutional objection, and how to navigate it, for the benefit of patients, practitioners, and the public. This could be achieved in a variety of ways. As a baseline, jurisdictions should enact laws that require institutions to publicly disclose their position on VAD. This should include the implications of that position on the VAD information (if any) that will be shared with patients and the clinical services that will be provided. Transparency can also be fostered by including more concrete information in existing public-facing policies to provide better guidance to patients/residents, families, health professionals, and the public. Ideally regulation should enable individuals to make decisions about their care and set out a process for how VAD requests will be approached. Even institutions that do not want to participate should consider providing a practical procedural approach in their public-facing policies to benefit patients, families, staff, and other health professionals.

Institutional objection to VAD is a contentious issue, which requires regulation to reconcile the rights and interests of institutions that do not wish to participate with those of patients seeking access to VAD. Significantly, eligible patients are likely to be in a more vulnerable position than the institution and this goes beyond the usual information and power asymmetry seen in healthcare; by definition in Victoria, such patients are terminally ill, have a limited life expectancy, and are suffering. The current law and policy position in Victoria has left several gaps and areas of uncertainty. To achieve greater protection for patients, and more transparent and consistent policies, a stronger regulatory response from the government and legislature is needed. Ideally, jurisdictions will pass laws that clarify the obligations of institutions, uphold the needs of patients, and curtail institutions’ ability to object in circumstances and ways that would cause patients harm (White et al. 2021).

## Footnotes

1 Some surveys suggest that a significant proportion (up to approximately 70%) of Catholics in Australia support VAD, though the authors note that public attitudes towards VAD are difficult to measure as they are influenced by framing effects and survey methodology (Cartwright and Douglas 2017).
